# The Public Health and the Duty of the Daily Press

**Published:** 1903-10-03

**Authors:** 


### The Public Health and the Duty of the Daily Press.

We believe that among the curiosities of the present age not the least interesting to future generations will be the history of preventative medicine. The story of the continual striving of a single profession, whose members may be said to subsist by the prevalence of disease, to educate unwilling masses to preserve their health, wealth and welfare by taking reasonable precautions, is indeed a matter for wonder. The present era will probably be regarded much in the same light by which we interpret the history of the great epidemic of secret poisoning which swept over Europe in the sixteenth and early seventeenth centuries, and which in the public mind is commonly associated with the name of Borgia. We have heard a good deal of astonishment expressed that matters could come to such a pass that killing a neighbour by poison was scarcely thought to be a crime, while murder by direct violence was looked upon with horror and punished with the utmost severity. But to the philosophic mind there is no need to journey back so far as the seventeenth century, or even to depart from the present time to behold an almost identical phenomenon. Do we not now without any manifestations of horror allow people to go their way, killing or maiming their fellows and even our own children with the subtle poisons of disease? Nor is it to infectious illnesses alone that this applies; many a preventable disease, for example, infantile diarrhœa, is allowed to scourge humanity through the neglect of comparatively simple precautions. The country loses millions of money, the nation pays its toll in thousands upon thousands of human lives every year, and yet no great abhorrence is aroused. The psychological explanation lies partly no doubt in custom, for it is this that robs men of fear and disgust. "Custom doth make dotards of us all." But the main barrier is popular ignorance.

To what purpose is it that medical men are trying to teach the elements of hygiene to the multitude? Theirs are voices crying in the wilderness, and will be so for many a long year to come unless the one means through which the public can be instructed, the daily press, recognises its responsibility in these matters, and sets to work on new lines accordingly. So far it would appear that the newspapers merely serve to increase public ignorance in medical matters, with regard to which it is really of the highest importance they should be well informed. It was only a short while ago that one of the leading London papers published a copyright cablegram announcing two very important new discoveries concerning cancer. It so fell out that of these two "new discoveries" one was twenty years old and the other more than ten to our certain knowledge, and what is also noteworthy is that any professional man very moderately conversant with medical progress could recognise the two "new discoveries" as mere scraps picked up off the rubbish heap of forsaken ideas. Indeed, one sees almost every day short notices in the morning paper which are supposed to convey some piece of medical intelligence, but which have evidently not been written by anyone having a knowledge of the subject. Such articles as these can only serve to disgust medical men, bewilder the public, and make the newspaper concerned appear ridiculous.

It is by no means our wish to abuse the great daily press, nor do we think that they are really callous towards the public health. But we do most honestly believe that the public is anxious for instruction which will enable it to back up the medical profession in its attempts to check the ravages of disease. The lay members of the community cannot read the medical journals with any advantage, because they are unable to follow the technical language; they therefore rely to a great extent upon the daily papers to act as their interpreters and to keep them informed of important facts in this as in other public matters. Surely it would not be a very difficult matter for the better class of newspaper to include a medical man among its staff. It is a great and important duty which the daily press has to perform, and what we most desire is to see it rise to the occasion, and by a careful supervision to educate its readers in matters which have a bearing on State medicine, and so pave the way for those legislative enactments for safeguarding the public health which at the present moment are so much to be desired.

